# MP2-IQA: upscaling the analysis of topologically partitioned electron correlation

**DOI:** 10.1007/s00894-018-3717-5

**Published:** 2018-07-11

**Authors:** Arnaldo F. Silva, Paul L. A. Popelier

**Affiliations:** 1 Instituto de Química, Universidade Estadual de Campinas, Campinas, SP 13083-970 Brazil; 2 Manchester Institute of Biotechnology, the University of Manchester, 131 Princess Street, Manchester, M1 7DN Great Britain; 3 School of Chemistry, the University of Manchester, Oxford Road, Manchester, M13 9PL Great Britain

**Keywords:** Electron correlation, FFLUX, Force field, Interacting quantum atoms (IQA), Quantum chemical topology (QCT)

## Abstract

When electronic correlation energy is partitioned topologically, a detailed picture of its distribution emerges, both within atoms and between any two atoms. This methodology allows one to study dispersion beyond its more narrow definition in long-range Rayleigh-Schrödinger perturbation theory. The interacting quantum atoms (IQA) method was applied to MP2/6-31G(d,p) (uncontracted) wave functions of a wide variety of systems: glycine…water (hydration), the ethene dimer (π-π interactions), benzene (aromaticity), cyclobutadiene (antiaromaticity), and NH_3_BH_3_ (dative bond). Through the study of molecular complexes it turns out that dispersion energy is either important to a system’s stabilization (for the C_2_H_4_ dimer) or not important (for Gly…H_2_O). We have also discovered that the delocalization in benzene lowers the strength of Coulomb repulsion in the bonds, which has been quantified for the first time through IQA. Finally, we showed that the nature of the dative bond is much different from that of a regular covalent bond as it is not destabilized by electronic correlation. Finally, the conclusions obtained for these archetypical systems have implications for the future of the quantum topological force field FFLUX in the simulation of larger systems.

**Electronic supplementary material:**

The online version of this article (10.1007/s00894-018-3717-5) contains supplementary material, which is available to authorized users.

**Graphical abstract:**
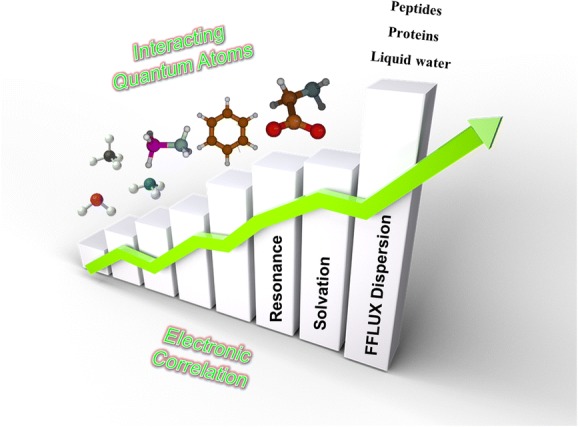
Atomic and bond dynamic correlation energies are now available thanks to IQA. Larger molecules can now be accessed to include resonance and solvation of FFLUX force field

## Introduction

Obtaining meaningful electronic correlation energy (ECE) and dispersion energy continues to be a priority in the electronic structure community. Density functional theory (DFT) methods can also benefit from the development of electronic correlation methods because dispersion corrections in DFT is a popular theme in the recent literature. Yet, reports on how electronic correlation affects different molecular regions in space, including intramolecular and intra-atomic contributions, are scarce. We have recently published a series of papers [1–4] that answer some of these questions, using quantum chemical topology [5–13] (QCT) through one of its tools called the interacting quantum atoms [14] (IQA) method. Among all other energy components, the IQA method enables a decomposition of electronic correlation, thereby permitting an exhaustive analysis of atoms and bonds in a molecular system. Our series of papers explored more than 50 small systems [2], including van der Waals complexes, radicals and ions, and focused mostly on MP2 correlation although higher orders of perturbation were also surveyed [3]. In that same paper, it was shown that bond correlation energies are rather stable over basis set changes; while moving from 6-31G(d,p) to 6-311G(2d,2p), only small changes are observed for bond correlations. The only instance in which a sign change occurs is for the HF molecule (−0.5 kJ mol^−1^ to 1.5 kJ mol^−1^), where the actual value is very small. The localization of ECEs by means of IQA shows that much correlation energy is stored in the atoms themselves, and hence the intra-atomic contribution dominates stabilization effects in molecules and their complexes. Investigating bond electronic correlation energies is more intricate than investigating atomic correlation energies because they are second in magnitude compared to intra-atomic correlation and their sign is sensitive to the nature of the bond. As far as our knowledge goes, it was only recently that anyone reported the ability to calculate ECEs for a MPn wave function within the IQA formalism. This capability was lacking for 10 years but was finally made possible through our work and our unique software. Furthermore, Pendás et al. [15] also carried out an IQA electron correlation analysis of several systems, but they used CCSD(T) densities. In the paper, a quick comparison between MP2 and CCSD(T) densities shows that the use of the unrelaxed MP2 densities can result in incomplete descriptions of the kinetic and electrostatic terms in the IQA partition, especially when compared to the relaxed CCSD(T) densities. Despite the differences between the classical contributions of MP2 and CCSD(T), very similar conclusions are reached when it comes to their exchange-correlation parts of the IQA partition.

In the current paper we make use of recently made advances in the computer program MORFI, which is a local modified version of MORPHY98 [16], in order to investigate larger systems. Amongst these advances are the implementation of an angular Laikov-Lebedev [17] quadrature, parallelization, and improved memory management. We ask this question: can MP2 correlation interpret the electronic phenomena that take place in these larger systems?

## Methods

### Partitioning methods: interacting quantum atoms

Several energy partitioning methods have been proposed in the literature over the last few decades. They are used as tools to extract chemical insight from systems by attributing physical meaning to their different energetic terms. Early methods, such as interference and valence partitioning [18], were successful in separating the total energy of a system into: (i) an unperturbed reference (quasi-classical or valence part) and (ii) a quantum mechanical interference (two-particle effects). These two terms became the foundation of many other partitioning schemes, including IQA. Over time more partitioning methods were developed. One of them, called distributed multipole analysis [19, 20], divides the electron density using the space of Gaussian basis functions, and is used to calculate electrostatic interactions between molecules. Energy partitioning has also been applied to obtain dispersive interactions between two separate molecules (fragments) by symmetry adapted perturbation theory (SAPT) [21]. However, dispersion within SAPT is only defined *between* these fragments as opposed to additionally *within a fragment*. Therefore, the dispersion interaction between the two termini of a U-shaped chain-like molecule, for example, experiences severe conceptual problems. A new electron density based partitioning scheme of interaction energies has been developed [22] to overcome some of SAPT’s shortcomings. This new partitioning method shares many similarities with SAPT, such as its first order contributions and its second order terms, which are analogous to the second order SAPT contributions and their exchange corrections.

However, it has been recognized that real space partitioning is more appropriated to divide density between atoms, which is proficiently done by IQA. The IQA decomposition is part of the overarching QCT methodology. An attractive feature of IQA is its ability to divide the total energy (E_total_) of a system into intra-atomic and interatomic terms, in an additive manner, and without invoking reference state. The total energy of the system can be written as a sum of IQA atomic energies:

1 $$ {E}_{total}=\sum \limits_A{E}_{IQA}^A=\sum \limits_A\left({E}_{\mathrm{intra}}^A+\frac{1}{2}\sum \limits_{B\ne A}{V}_{\mathrm{inter}}^{AB}\right)=\sum \limits_A{E}_{\mathrm{intra}}^A+\frac{1}{2}\sum \limits_A\sum \limits_{B\ne A}{V}_{\mathrm{inter}}^{AB} $$

where the intra-atomic energy can be expanded as:

2 $$ {E}_{\mathrm{intra}}^A={T}^A+{V}_{ee}^{AA}+{V}_{en}^{AA} $$

where *T*^*A*^ is the kinetic energy of the electrons in the volume of atom *A*, ${V}_{ee}^{AA}$ the intra-atomic electron-electron potential energy, and ${V}_{en}^{AA}$ the intra-atomic electron-nuclear potential energy. Note the generality of these equations as they are valid in whichever system the atoms appear, independent of where (strongly) covalent interactions occur.

We have stated before that some of the most interesting phenomena can be observed in the interatomic potential energy, which can be written as:

3 $$ {V}_{\mathrm{inter}}^{AB}=\left({V}_{nn}^{AB}+{V}_{en}^{AB}+{V}_{ne}^{AB}\right)+{V}_{ee}^{AB} $$

where ${V}_{nn}^{AB}$ is the inter-nuclear repulsion energy between the nuclei of atoms *A* and *B*, while ${V}_{ee}^{AB}$ is the interatomic electron-electron potential energy. The ${V}_{ee}^{AB}$ energy term encompasses Coulomb, exchange, and correlation potentials, and can be written as:

4 $$ {V}_{\mathrm{ee}}^{AB}={V}_{ee, coul}^{AB}+{V}_{ee, exch}^{AB}+{V}_{ee, corr}^{AB} $$

where the three terms on the right hand side respectively refer to the Coulomb, exchange, and correlation parts of the electron-electron potential energy. The last term is the focal point of our analysis, where atoms *A* and *B* are not necessarily bonded but can be at any distance from each other, allowing for the quantitative description of so-called through-space effects.

Note that the partitioning schemes discussed above, including IQA, seek to derive physical meaning from the energetic contributions given by their equations and integrals. Some terms, such as Coulomb, exchange, and dispersion, are common to most partitioning schemes but they do not represent the same quantities and must be compared with caution. However, there are instances in which terms of some partitioning methods can have a similar interpretation to the equivalent of IQA (for a full example of comparison, see ref. [23]). This is the case for the electrostatic term of Morokuma’s partition scheme [24] and IQA’s Coulomb interaction: both characterize moieties interacting through classic Coulombic forces. Nonetheless, the two schemes diverge when it comes to their quantum effects: the exchange-correlation part of the IQA decomposition represents quantum overlap and the actual electronic correlation in a system, and the polarization-dispersion part of Morokuma’s decomposition scheme is simply a polarization effect experienced by one molecule in the presence of the other. Furthermore, IQA uses real space partitioning, which makes it conceptually different from the traditional partitioning methods described above.

### IQA electronic dynamic correlation energy

The details of our approach have been exhaustively explained in our previously mentioned series of publications but here we repeat the key equation:

5 $$ {V}_{ee, corr}^{AB}=\sum \limits_{j=1}^{N_G}\sum \limits_{k=1}^j{k}_{jk}\sum \limits_{l=1}^{N_G}\sum \limits_{m=1}^l{k}_{lm}{d}_{jk lm}^{corr}{\int}_{\Omega_A}d{\mathbf{r}}_1{G}_{jk}\left({\mathbf{r}}_1-{\mathbf{R}}_{jk}\right){\int}_{\Omega_B}d{\mathbf{r}}_2\frac{1}{r_{12}}{G}_{lm}\left({\mathbf{r}}_2-{\mathbf{R}}_{lm}\right) $$

where *N*_*G*_is the number of primitive Gaussian basis functions *G*_*pq*_, Ω is the volume of an atom, and the two-particle density matrix (2PDM) (with elements ${d}_{jklm}^{corr}={d}_{jklm}^{MP2}-{d}_{jklm}^{Hartree- Fock}$) is the result of removing the Hartree-Fock contributions to the MP2 2PDM. Note that the two 3D volume integrals in Eq. (5) are coupled because the inter-electronic distance r_12_ depends both on **r**_1_ and **r**_2_. The interaction energy (V) of each atom with itself (A = B) or with one of the other atoms (A ≠ B) is obtained from the 2PDM via a 6D quadrature integration.

### Computational details

In our methodology, we choose to deliberately separate the correlated part of the two-particle density matrix in order to evaluate two-particle effects independently from one-particle effects. The correlated wave functions and density matrices are first obtained by a default Gaussian geometry optimization and single point calculation, so we can extract the correlated part of the 2PDM from Gaussian09 [25] (Link1111 and associated routines). The correlated part of the 2PDM is converted into the primitive basis format to then be used by a locally written version of MORPHY98 called MORFI. The orbitals, geometries, and primitive basis sets are obtained from Gaussian09 via the standard wave function file (*.wfn format). In this step we obtain uncorrelated wave functions and orbitals, so we opt not to use the keyword “density = current” in the Gaussian09 input (resulting in Hartree-Fock densities). Since, in our methodology, the Møller-Plesset perturbation theory correlates only the two-electron energy terms of the Hartree-Fock (HF) theory, and we extracted those terms from the two-particle density matrix, the one-electron energy terms are actually identical to their HF counterparts. This means that correlation is only considered for the electron-electron Coulomb and exchange terms, which are in the correlated part of the 2PDM. MORFI topologically divides our systems into atoms and then uses these atoms to integrate the 2PDM via the ${d}_{jklm}^{corr}$ elements to yield intra-atomic (A = B) and interatomic (A ≠ B) correlation energies. Therefore, at the end of the calculation, our local code (MORFI) obtains the electronic correlation term (Eq. 5), as well as the kinetic, Coulomb, and exchange terms. The sum of all these energy terms results in the total electronic energy, which would be numerically identical to the one obtained from Gaussian09, if the atomic integration error were zero.

All the calculations were carried out at the MP2/6-31(d,p) level of theory, including geometric optimizations and electronic correlation calculations. We are aware that, due to the multiconfigurational character of benzene, the MP2-corrected Hartree-Fock reference is not adequate to supply an adequate wave function, but we still believe that it has the general correct form. The associated calculations are expensive and their cost depends on the number of primitive basis functions and the size of the quadrature grid used. Here we used 194 angular Lebedev-Laikov quadrature grid points and 20 G-Legendre radial grid points for all atoms in all systems. The total number of grids points per atom is 3880, which is only about a quarter of the number used for oxygen in our water cluster analysis [4]. It is the large computational cost of the MP2-IQA analysis that economizes the quadrature grid. However, when comparing the original electron correlation energy calculated by Gaussian09 with that reconstructed from the IQA contributions the maximum discrepancy found is 13 kJ mol^−1^ (for the ethene dimer system).

## Results

### Van der Waals complexes

#### The role of electronic correlation in the hydration of glycine

**Fig. 1:**
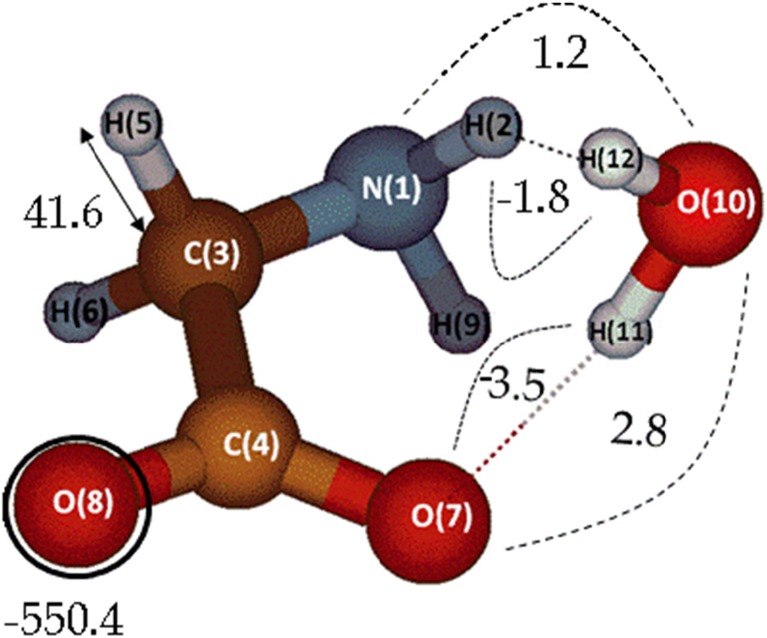
Optimized geometry of deprotonated glycine complexing with a single water molecule. A few examples of the three different types of ECEs are highlighted: an oxygen’s atomic correlation energy (encircled), a C-H bond correlation energy (indicated by a double arrow), and some hydrogen bond interactions (marked by dashed lines), such as H(2)….O(10)

We tested for the first time, using MP2-IQA, the effects of electronic correlation on the hydration of deprotonated glycine (in a basic environment, as is the case in human blood) while interacting with a single water molecule. Figure 1 shows the glycine…water complex studied here. Future work should focus on peptide hydration by adding many more water molecules, but here we make a start in breaking away from pure water clusters or an amino acid in the gas phase.

The overall error in the reconstruction of the total electronic correlation obtained by Gaussian09 (i.e., original or “exact” energy) was 5.1 kJ mol^−1^, which corresponds to 0.2% of the grand total correlation energy. This error is caused by the limitations of numerical integration algorithm. Table 1 is divided in three major sections: atomic correlation energies, bond correlation energies, and nonbonded interactions. A few observations can be made regarding the absolute magnitude and signs of these energy components. The atomic correlation energies correspond to the largest portion of the total correlation energy, followed by bond correlation (which can be about ten times smaller than atomic correlation for the heavier atoms, non-hydrogens). Nonbonded correlation interactions are the least relevant contributions energetically, being about 20 times smaller than bond correlation energies.

**Table 1:** Electron correlation energies (ECEs) (kJ mol^−1^) for the glycine…water complex. Only ECEs larger than 1.0 kJ mol^−1^ are shown here. All nonbonded intramolecular ECEs smaller than this threshold are added up together under the label “other intramolecular”. Similarly, nonbonded intermolecular ECEs have been added and reported under “other intermolecular”

Type	Label	Atom or interaction	Correlation energy (kJ mol^−1^)
Atomic	1	N	−481.4
Atomic	2	H	−28.5
Atomic	3	C	−351.4
Atomic	4	C	−205.5
Atomic	5	H	−37.7
Atomic	6	H	−37.7
Atomic	7	O	−555.4
Atomic	8	O	−550.4
Atomic	9	H	−29.7
Atomic	10	O	−489.9
Atomic	11	H	−13.6
Atomic	12	H	−21.9
Total atomic			−2803.1
Bond	1-2	N-H	36.4
Bond	1-9	N-H	38.8
Bond	1-3	C-N	28.3
Bond	3-4	C-C	29.9
Bond	3-5	C-H	41.6
Bond	3-6	C-H	41.7
Bond	4-8	C=O	10.1
Bond	4-7	C=O	3.5
Bond	10-12	O-H	21.4
Bond	10-11	O-H	10.0
Total bond			261.6
Nonbonded^a^	8...7	O...O	11.6
Nonbonded^a^	8...3	C...O	6.5
Nonbonded^a^	7...3	C...O	5.4
Nonbonded^a^	4...1	N...C	3.7
Nonbonded^a^	6...5	H...H	−5.2
Nonbonded^a^	9...2	H...H	−4.8
Nonbonded^a^	8...5	O...H	−3.5
Nonbonded^a^	8...6	O...H	−3.5
Nonbonded^a^	12...11	H...H	−2.7
Nonbonded^a^	9...7	O...H	−2.6
Nonbonded^a^	5...2	H...H	−2.1
Nonbonded^a^	9...6	H...H	−1.8
Nonbonded^a^	7...2	O...H	−1.2
Nonbonded^a^	9...4	C...H	−1.2
Nonbonded^a^	6...1	N...H	−1.1
Nonbonded^a^	9...3	C...H	−1.1
Other^a^ intramolecular			−6.1
Hydrogen bond^b^	11...7	O…H	−3.5
Hydrogen bond^b^	10...2	O...H	−1.8
Hydrogen bond^b^	10...7	O...O	2.8
Hydrogen bond^b^	10...1	N...O	1.2
Other^b^ intermolecular			−3.0
Total intra			−9.7
Total inter			−4.3
Total^c^			−14.0

a) Intramolecular

b) Intermolecular

c) Intramolecular + Intermolecular

The atomic correlation energies are always negative. However, bond correlation is always positive for the covalent bonds in glycine’s internal structure, within the complex. It must be pointed out that there are some instances in which bond correlation can be negative, as we reported elsewhere [2] for some ionic bonds and most notably hydrides. In fact, Pendás et al. [15] also reported the negative exchange-correlation components in the hydride BH_3_, justifying this observation by the poor Hartree-Fock description of the hydride-like atomic volume. The sign of the bond correlation energy is an indication of bond polarity: low polarity bonds have large positive bond correlation energies (the electronic density is concentrated at the interatomic region) because high polarity bonds have small or even negative bond correlations (the electronic density is concentrated at the atomic basins). However, it is important to highlight that even in the cases in which bonds have highly positive correlation energies, the values are largely stabilized by even more negative exchange energies. For example, the N(1)-H(2) bond, in Table 1, has a correlation energy of 36.4 kJ mol^−1^ and an exchange energy of −340.5 kJ mol^−1^.

Lastly, the nonbonded interactions are abundant and mainly negative. The most negative of these nonbonded interactions are the H…H interactions, generally around −5.0 kJ mol^−1^. The notable exceptions are interactions between heavy (O, N or C) atoms, which can be surprisingly large, such as the O(7)…O(8) correlation (11.6 kJ mol^−1^), which is about the same value as that of the C(4) = O(8) bond (10.1 kJ mol^−1^). The complete list of values can be found in Table S1 in the Supporting information (SI).

As can be seen in Table 1, the terms involved in the hydrogen bond are rather large, especially compared to an average nonbonded term. In fact, the terms that are directly involved in the hydrogen bonds are the only intermolecular interactions that have absolute values larger than or equal to 1.2 kJ mol^−1^ (based on inspection of Table S1). Furthermore, the O…H hydrogen bond ECEs are negative but the O…O and O…N ECEs are positive, causing a partial cancelation in the correlation energy of the hydrogen bond, taken here to be the three-atom system [O-H…O] or [N-H…O]. This cancellation was already observed in a study of electronic correlation in water clusters [4], where we reported that electronic correlation plays only a minor role in hydrogen bonding. Adding the four hydrogen bond interactions mentioned above yields only −1.3 kJ mol^−1^ due to the nearly perfect cancellation of positive and negative values. Furthermore, adding all the interactions between the water and glycine (not only the interactions directly involved in the hydrogen bond but indeed all the intermolecular terms) yields a total ECE of only −4.3 kJ mol^−1^. These results point to the fact that correlation energies (i.e., dispersion) play only a minor role in the hydration of amino acids, as most of the stabilization is of a Coulomb and exchange nature.

#### The π-π interaction of the ethene dimer (C_2_H_4_…C_2_H_4_)

**Fig. 2:**
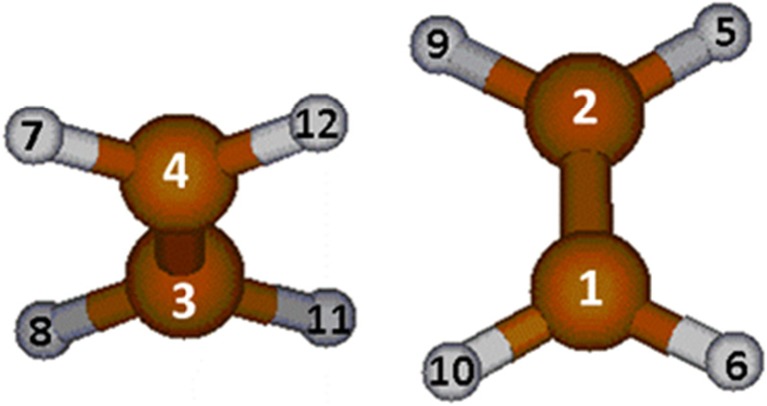
Optimized geometry of the D_2d_ configuration of the ethene dimer, reported by several sources as the most stable one

In contrast to the Gly…H_2_O case described above, the ethene dimer (C_2_H_4_)_2_ is a weakly bound complex formed by a molecule with a vanishing permanent electric dipole moment. However, both systems share a couple of similarities, such as the total number of atoms being 12 in each system, and the fact that the systems are highly simplified archetypes of biologically important interactions. Indeed, weak nonbonding π-π interactions are known [26] to be a determinant factor in the structure of nucleic acids and DNA. The C_2_H_4_…C_2_H_4_ complex is the simplest case representing such an interaction. Actually, there are over a dozen configurations for the ethene dimer [27], and the relative energy between these geometries are tempered by a complex balance between London’s dispersion forces and electrostatic quadrupole-quadrupole interactions. Over the years, several studies have being carried out attempting to pinpoint the most stable configuration for the ethene dimer [28–30]. These studies agree that the D_2d_ configuration, as shown in Fig. 2, is the most stable one, and therefore it was selected.

The error in the numerical integration found for the dimer was 7.0 kJ mol^−1^, which represents about 0.5% of the total correlation energy. Table 2 shows the relevant ECEs organized in the same way as in Table 1 for hydrated glycine. All the actual numbers can be found in Table S2. Note that nonbonded interactions can again be sub-categorized in intramolecular interactions and intermolecular interactions.

**Table 2:** Electron correlation energies (ECEs) (kJ mol^−1^) for the ethene dimer. Only ECEs larger than 1.0 kJ mol^−1^ are shown here. All nonbonded intramolecular ECEs smaller than this threshold are omitted but added together to the ones that are larger than 1.0 kJ mol^−1^ under “total intramolecular”. There were no nonbonded intermolecular ECEs larger than 1.0 kJ mol^−1^ and their sum can be seen in “total intermolecular”

Type	Label	Atom or interaction	Correlation energy (kJ mol^−1^)
Atomic	1	C	−413.9
Atomic	2	C	−413.9
Atomic	3	C	−413.8
Atomic	4	C	−413.9
Atomic	5	H	−35.5
Atomic	6	H	−35.5
Atomic	7	H	−35.5
Atomic	8	H	−35.5
Atomic	9	H	−35.5
Atomic	10	H	−35.5
Atomic	11	H	−35.5
Atomic	12	H	−35.5
Total atomic			−1939.2
Bond	1-2	C-C	142.5
Bond	3-4	C-C	142.6
Bond	5-2	C-H	38.2
Bond	6-1	C-H	38.2
Bond	9-2	C-H	37.5
Bond	10-1	C-H	37.5
Bond	7-4	C-H	38.2
Bond	8-3	C-H	38.2
Bond	12-4	C-H	37.5
Bond	11-3	C-H	37.5
Total bond			587.9
Nonbonded^a^	5...1	C...H	−1.3
Nonbonded^a^	9...1	C...H	−1.0
Nonbonded^a^	6...2	C...H	−1.3
Nonbonded^a^	10...2	C...H	−1.0
Nonbonded^a^	7...3	C...H	−1.4
Nonbonded^a^	12...3	C...H	−1.0
Nonbonded^a^	6...4	C...H	−1.3
Nonbonded^a^	11...4	C...H	−1.0
Nonbonded^a^	9...5	H...H	−4.2
Nonbonded^a^	10...6	H...H	−4.2
Nonbonded^a^	12...7	H...H	−4.2
Nonbonded^a^	11...8	H...H	−4.2
Nonbonded^a^	6...5	H...H	−2.8
Nonbonded^a^	10...9	H...H	−2.6
Nonbonded^a^	12...11	H...H	−2.6
Nonbonded^a^	8....7	H...H	−2.8
Nonbonded^a^	9...6	H...H	−1.0
Nonbonded^a^	10...5	H...H	−1.0
Nonbonded^a^	11...7	H...H	−1.0
Nonbonded^a^	12...8	H...H	−1.0
Total intramolecular			−41.2
Total intermolecular			−2.8
Total Nonbonded^b^			−44.0

a) Intramolecular

b) Intramolecular + intermolecular

As expected, we see again that most of the total correlation energy originates from the atomic correlation, which is slightly counteracted by positive bond correlation energies and accompanied by a significantly smaller negative contribution originated from nonbonded interactions. So far, these observations are, up to a point, the same ones explained in connection with Gly...H_2_O. However, when it comes to the nonbonded intermolecular interactions, there are some major differences.

First, there are no intermolecular interactions larger than 1.0 kJ mol^−1^ in C_2_H_4_…C_2_H_4_, as was also observed for Gly…H_2_O. The largest intermolecular interactions for the ethene dimer occur between the closest hydrogen atoms (12 and 10, in Fig. 2, for example), with a value of −0.7 kJ mol^−1^ (not in Table 2). Furthermore, there are only a few positive intermolecular ECEs, which are not large enough to cancel the small stabilization that originates from the negative ECEs. This means that the cancellation between positive and negative interactions observed for ECEs in the hydrogen bond (glycine…water case) does not occur in the C_2_H_4_…C_2_H_4_ complex. The net value of the intermolecular ECEs is only −2.8 kJ mol^−1^, which is small, but roughly of the same magnitude as the total stabilization energy of the ethene complex, −6.5 kJ mol^−1^, calculated at the MP2/CBS level of theory [31].

#### Benzene and cyclobutadiene: the role of electronic correlation in aromaticity and antiaromaticity

**Fig. 3:**
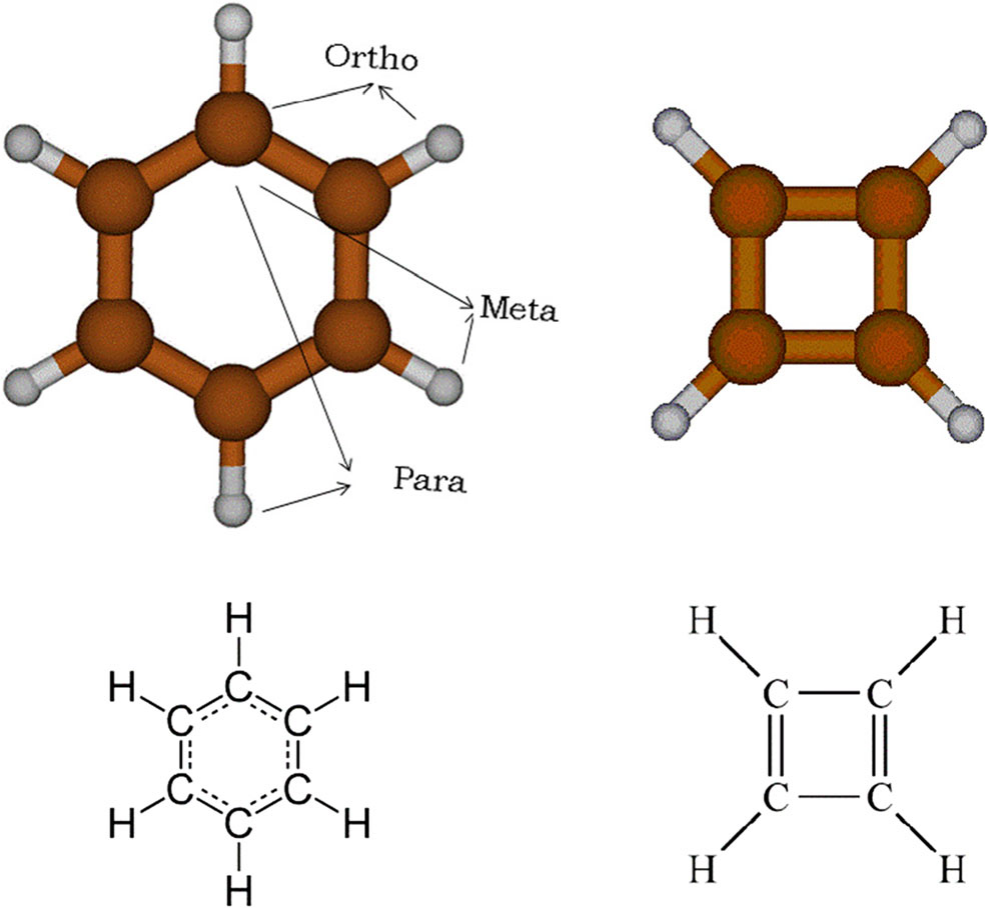
Optimized geometry of benzene and cyclobutadiene, and the representation of their delocalized and localized bonds, respectively. Some examples of C…H interactions are marked in benzene in order to better understand entries in Table 3

Here we look at benzene and cyclobutadiene as prototypes of aromatic and antiaromatic systems, respectively, as shown in Fig. 3. The IUPAC’s gold book [32] defines these systems in the following way: “*A cyclically conjugated molecular entity with a stability (due to delocalization) significantly greater than that of a hypothetical localized structure (e.g. Kekulé structure) is said to possess aromatic character. If the structure is of higher energy (less stable) than such a hypothetical classical structure, the molecular entity is ‘anti-aromatic’*”.

Although benzene has been a specific subject of study (e.g., the influence of quantum mechanical interference on its geometric features [33] or the origin of exalted frequency is its first excited state [34]), there appear to be no studies that focus on understanding how electronic correlation effects (dispersion and polarization) affect aromaticity and resonance at a real-space-partitioned atomic level. In the current work, we focus on the following key chemical concepts: stability and delocalization. Is IQA-partitioned electronic correlation capable of differentiating between aromaticity and antiaromaticity?

#### Benzene, electron delocalization, and aromaticity

We analyze Table 3 first. The error in the reconstruction of the total electronic correlation obtained by Gaussian was 13.0 kJ mol^−1^, which is reasonably larger than the error observed for Gly…H_2_O and the ethene dimer, but still corresponds to only 0.6% of the total correlation energy. Table 3 is divided similarly to Tables 1 and 2: atomic correlation energies, bond correlation energies, and nonbonded interaction energies.

**Table 3:** Electron correlation energies (ECEs) (kJ mol^−1^) for benzene. The total energy for the electronic correlation of benzene can be obtained by multiplying the ECE value of each identical atom or interaction by the number of occurrences

Type	Atom or interaction	Number of occurrences	Correlation energy (kJ mol^−1^)
Atomic	C	6	−406.4
Atomic	H	6	−48.4
Total atomic			−2728.9
Bond	C-C	6	62.2
Bond	C-H	6	34.2
Total bond			578.6
Nonbonded	C...C	Meta 6	10.2
Nonbonded	C...C	Para 3	6.6
Nonbonded	C...H	Ortho 12	−0.1
Nonbonded	C...H	Meta 12	−0.2
Nonbonded	C...H	Para 6	−0.6
Nonbonded	H...H	Ortho 6	−2.8
Nonbonded	H...H	Meta 6	−0.3
Nonbonded	H...H	Para 6	−0.5
Total nonbonded			52.3

The rationale behind the positive value for the ECE associated with the CC bond is that, compared with the Hartree-Fock reference, the MP2 densities take into account antiparallel (opposite spin) Coulomb electron correlation. This fact will cause repulsion between electrons, especially in low-polarity covalent bonds. This effect is naturally enhanced by multiple bonds, which contain a larger electron density. This observation is supported by the ascending order of the bond correlation energies in the series [2] of F_2_ (single bond, 86.9 kJ mol^−1^), O_2_ (double bond, 292.3 kJ mol^−1^) and N_2_ (triple bond, 400.1 kJ mol^−1^). In other words, the electronic correlation energies shown here do not necessarily represent dispersion. The positive values observed at most bonds do not correspond to what is described as a dispersive interaction, i.e., small and stabilizing. We can infer that only for larger atomic distances can electronic correlation energies be interpreted as dispersion, at short distances ECEs are actually a measurement of electronic repulsion.

**Fig. 4:**
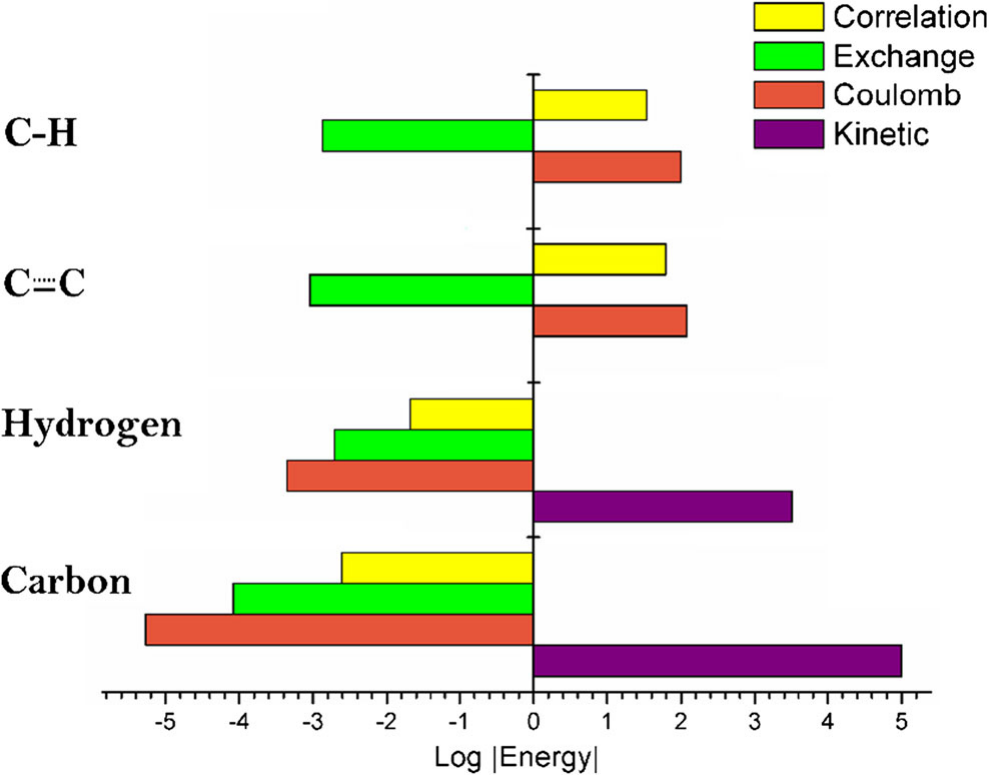
Complete IQA fingerprint of carbon, hydrogen, and the CC and CH bonds in benzene. Negative energies appear on the left, as the original signs are reintroduced after the log operation; for example, hydrogen’s Coulomb energy is about −2250 ≈ −10^3.4^ kJ mol^−1^

Note that although the electronic correlation energy can add up (over a whole system) to several thousands of kJ mol^−1^ (for example, intra-atomic contribution see Table 3), it is not necessarily the largest contribution to the total energetic signature of an atom or a bond. In fact, sometimes the electronic correlation energy is just a small part of the total energy. Indeed, there are other IQA terms, such as exchange and Coulomb, which are typically much larger. To illustrate this fact, in Fig. 4 we diagrammatically show a breakdown of the energy contributions of carbon, hydrogen, and the CC and CH bonds in benzene.

#### Cyclobutadiene and antiaromaticity

For cyclobutadiene, the integration error for the total electronic correlation obtained by MORFI is significantly smaller than that for benzene, and amounts to only 3.5 kJ mol^−1^. Cyclobutadiene, unlike benzene, is an antiaromatic molecule. The bond correlation energy analysis is a good probe for this property, as the CC bonds fall in two different categories: single (29.8 kJ mol^−1^) and double (159.9 kJ mol^−1^) bonds, as shown in Table 4. Note that the bonds in benzene, considered to have a bond order of 1.5, have a bond ECE falling in-between the two types of CC bonds in cyclobutadiene.

**Table 4:** Electron correlation energies (ECEs) (kJ mol^−1^) for cyclobutadiene. The total energy for the electronic correlation of cyclobutadiene can be obtained by multiplying the ECE value of each identical atoms or interaction by the number of occurrences

Type	Atom or interaction	Number of occurrences	Correlation energy (kJ mol^−1^)
Atomic	C	4	−430.6
Atomic	H	4	−35.0
Total atomic			−1862.3
Bond	C-C	2	29.8
Bond	C=C	2	159.9
Bond	C-H	4	37.1
Total bond			527.9
Nonbonded	C...C (1...3)	2	10.1
Nonbonded	C...H(1...3, double)	4	−1.1
Nonbonded	C...H(1...3, single)	4	−1.4
Nonbonded	C...H (1...4)	4	−0.3
Nonbonded	H...H(1,3 double)	2	−2.0
Nonbonded	H...H(1,3, single)	2	−1.7
Nonbonded	H...H(1-4)	2	−0.8
Total nonbonded			0.0

#### Comparing stability and delocalization of benzene and cyclobutadiene

So far we have only discussed electronic delocalization for the CC bonds. From this point onward the stabilization of these molecules will be addressed as well. Analyzing the total atomic correlation of benzene (third line, last column of Table 3) we obtain the value of −2728.9 kJ mol^−1^. If we divide this value by the number of atoms in benzene we obtain −2728.9/12 or − 227.4 kJ mol^−1^ of stabilization energy per atom (taking carbon and hydrogen atoms into account). The same quantity for cyclobutadiene is −1862.3/8 = −232.8 kJ mol^−1^ of stabilization per atom (third line, last column of Table 4). We can state that the stabilization stemming from atomic energies differs only slightly between the two systems.

If we carry on a similar analysis for bond correlation (sixth and seventh lines of Tables 3 and 4, respectively) we retrieve 578.6/12 = 48.2 kJ mol^−1^ destabilization per bond for benzene (12 bonds, considering CC and CH bonds) and 527.9/8 = 66.0 kJ mol^−1^ destabilization per bond for cyclobutadiene (8 bonds, total). As we stated before, the positive value for bond correlation indicates electronic repulsion, which is aggravated by multiple bonds. Since the CH bond correlation is very similar in both systems (34.2 and 37.1 kJ mol^−1^) the difference lies mostly in the CC bonds. Due to the delocalized nature of the electrons in the CC bonds of benzene, the burden of Coulomb electron correlation is distributed over a larger interatomic volume, diminishing the repulsion effect. This effect does not occur in cyclobutadiene because it is a localized system. This localized repulsion in cyclobutadiene is especially noticeable for the C=C double bond, which possess a bond correlation of 159.9 kJ mol^−1^. This means that the largest contributions to the stabilization of benzene come from the stabilization of its highly electronically populated conjugated bonds.

These results show that the IQA correlation energy method is not only capable of identifying both stability and delocalization in aromatic and antiaromatic systems but also that both concepts are intertwined. We can now answer the question we asked before; IQA partition is not only capable of differentiating aromaticity and antiaromaticity, but also of quantifying the stabilization provided by resonance: each bond in benzene is, on average, 17.8 (66.0 - 48.2) kJ mol^−1^ more stable than in cyclobutadiene.

#### Electronic B-N bond correlation in the donor−acceptor complex H_3_N:BH_3_

**Fig. 5:**
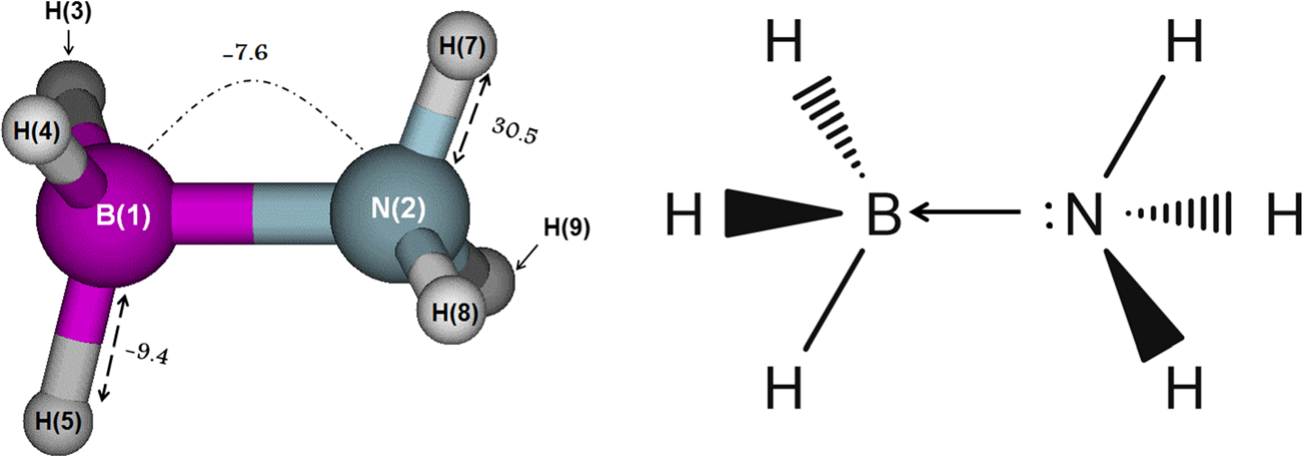
Optimized geometry of H_3_NBH_3_. A few examples of the most important ECEs are highlighted: the B-H and N-H bond correlation energies are indicated by a black arrow and the B-N bond correlation is marked by a dashed line

Finally we investigated the NH_3_BH_3_ molecule (see Fig. 5), which is one of the simplest and most well-known donor-acceptor complexes, also known as a charge-transfer complex. The idea behind this section is to get insight into the nature of the N-B interaction (or would it be N…B?) based on electronic correlation bond energies only. For this complex, the error in the total correlation energy was found to be outstandingly small, only 0.5 kJ mol^−1^, again solely due to numerical integration errors.

We first discuss bond correlation energies because the atomic energies do not display any special features. Table 5 shows all the ECEs for this molecule.

**Table 5:** Electron correlation energies (ECEs) (kJ mol^−1^) for NH_3_BH_3_

Type	Label	Atom or interaction	Correlation energy (kJ mol^−1^)
Self	1	B	−55.5
Self	2	N	−496.0
Self	3	H	−58.1
Self	4	H	−58.1
Self	5	H	−58.1
Self	6	H	−30.9
Self	7	H	−30.9
Self	8	H	−30.9
Total self			−818.4
Bond	1-3	B-H	−9.4
Bond	1-4	B-H	−9.4
Bond	1-5	B-H	−9.4
Bond	2-6	H-N	30.5
Bond	2-7	H-N	30.5
Bond	2-8	H-N	30.5
Total bond			63.4
Nonbonded	1...2	B...N	−7.6
Nonbonded	4...2	N...H	1.1
Nonbonded	3...2	N...H	1.1
Nonbonded	5...2	N...H	1.1
Nonbonded	6...1	B...H	0.2
Nonbonded	7...1	B...H	0.2
Nonbonded	8...1	B...H	0.2
Nonbonded	3...5	H...H (BH)	−1.3
Nonbonded	3...4	H...H (BH)	−1.3
Nonbonded	4...5	H...H (BH)	−1.3
Nonbonded	6...7	H...H (NH)	−4.1
Nonbonded	¨6...8	H...H (NH)	−4.1
Nonbonded	7...8	H...H (NH)	−4.1
Nonbonded	3...6	H...H	0.0
Nonbonded	3...7	H...H	−1.6
Nonbonded	3...8	H...H	−1.6
Nonbonded	4...6	H...H	−1.6
Nonbonded	4...7	H...H	−1.6
Nonbonded	4...8	H...H	0.0
Nonbonded	5...6	H...H	−1.6
Nonbonded	5...7	H...H	0.0
Nonbonded	5...8	H...H	−1.6
Total nonbonded			−30.0

Regarding the covalent bonds in the complex, N-H has a positive bond correlation energy of 30.5 kJ mol^−1^, while B-H has a negative bond correlation of −9.4 kJ mol^−1^. The moderate positive bond correlation of N-H is common for polar covalent bonds, and the small negative bond correlation of B-H is characteristic of the hydridic bond. The third bond (B-N) in the complex is much more difficult to pinpoint, and it is often referred to as a coordinate covalent bond or dative bond. The B-N dative bond distance is 1.660 Å, which it is too short for a nonbonded interaction. Its bond correlation energy is −7.6 kJ mol^−1^, which is nearly as negative as that of the B-H bond. In fact we calculated the bond correlation for the BN (triple bond) molecule at the same level of theory used in this article (MP2/6-31G(d,p)) for comparison’s sake, and its value was 229.9 kJ mol^−1^ at a bond distance of 1.327 Å. The observation that the difference between the B-N bond distances in BN and NH_3_BH_3_ are not that large (0.333 Å), and that the atoms involved are the same, suggests that the huge difference in the bond correlation energies is due to the very nature of those bonds. The BN molecule contains a triple bond, which is reflected by the very large bond correlation, but the B-N bond in NH_3_BH_3_ is unique in nature: it is the product of an interaction between a lone electron pair of the nitrogen atom and an empty (virtual at the ground state) orbital of boron.

The nature of the dative bond is responsible for its somewhat unusually large negative bond correlation energy, as, by definition, it involves a virtual orbital that is notoriously poorly represented by Hartree-Fock. It is natural to infer that MP2 would correct this shortcoming by adding correlation to this bond, making it more stable than the Hartree-Fock counterpart would predict.

## Conclusions

The role of electronic correlation (and what it physically represents) can change dramatically depending on the system and on which region of the molecule it is acting upon. When correlation occurs between directly bonded atoms, it usually acts as Coulomb repulsion, resulting in a positive correlation value, with some notable exceptions being the hydride bond and also the dative bond in BH_3_NH_3_. When electronic correlation is located between two nonbonded atoms it appears to act as classical dispersion, with small, stabilizing values.

Secondly, electronic correlation can be very important for the stabilization of some molecules, as was the case for CC in benzene and the ethene dimer, but there are cases where the overall contribution is partially cancelled, as seen in the glycine…water complex.

These MP2 results point to the fact that correlation energies (dispersion) play only a minor role in the hydration of amino acids, as most of the stabilization is Coulomb-like and exchange-like in nature. This effect is not due to the lack of dispersion forces acting between the molecules, but due to a large cancellation between small positive and negative correlation energies between them.

From our brief excursion into Gly…H_2_O and the ethene dimer we can conclude the following two statements, which we hypothesize can hold for larger biomolecular systems:

1. For Gly…H_2_O the intermolecular dispersion energies are actually rather large for hydrogen bond interactions but these specific pairwise interactions tend to cancel one another. Hence, even in a protein encased by a solvation shell, these numerous dispersion interactions will add up to a significant dispersion contribution.
2. For the C_2_H_4_…C_2_H_4_ complex the pairwise dispersion energies are indeed small but tend to add up quickly, reinforcing each other. In the case of a large molecule, such as DNA, these small contributions could end up playing a significant structural role.

## Electronic supplementary material

ESM 1 (DOCX 27 kb)
