# Supplementary material for: MP2-IQA: upscaling the analysis of topologically partitioned electron correlation
Source: J Mol Model. 2018 Jul 11;24(8):201. doi: 10.1007/s00894-018-3717-5 (PMC6061063; doi:10.1007/s00894-018-3717-5)
Supplement: Supplementary file 1 — (DOCX 27 kb) [file 894_2018_3717_MOESM1_ESM.docx]

**Supporting Information**

MP2-IQA: Upscaling the Analysis of Topologically Partitioned Electron Correlation

Arnaldo F. Silva^1^ and Paul L. A. Popelier^2^

^1^Instituto de Química, Universidade Estadual de Campinas, 13083-970, Campinas, SP, Brazil

^2^Manchester Institute of Biotechnology (MIB), 131 Princess Street, Manchester M1 7DN, Great Britain

and

School of Chemistry, University of Manchester, Oxford Road, Manchester M13 9PL, Great Britain

**Table S1**. Electron correlation energies (ECEs) (kJ mol^-1^) for glycine hydrated with one water molecule.

| Distance | Label | Atom or interaction | Correlation Energy (kJ mol^-1^) |
| --- | --- | --- | --- |
| Atomic | 1 | N | -481.37 |
| Atomic | 2 | H | -28.49 |
| Atomic | 3 | C | -351.43 |
| Atomic | 4 | C | -205.46 |
| Atomic | 5 | H | -37.70 |
| Atomic | 6 | H | -37.73 |
| Atomic | 7 | O | -555.40 |
| Atomic | 8 | O | -550.39 |
| Atomic | 9 | H | -29.71 |
| Atomic | 10 | O | -489.93 |
| Atomic | 11 | H | -13.60 |
| Atomic | 12 | H | -21.92 |
| Total atomic |  |  | -2803.13 |
| Bond | 1-2 | N-H | 36.42 |
| Bond | 1-3 | C-N | 28.34 |
| Bond | 3-4 | C-C | 29.87 |
| Bond | 3-5 | C-H | 41.56 |
| Bond | 3-6 | C-H | 41.67 |
| Bond | 4-8 | C=O | 10.08 |
| Bond | 4-7 | C=O | 3.45 |
| Bond | 9-1 | N-H | 38.78 |
| Bond | 10-12 | O-H | 21.37 |
| Bond | 10-11 | O-H | 10.00 |
| Total bond |  |  | 261.55 |
| Non-bonded | 2...3 | C...H | -0.79 |
| Non-bonded | 4...1 | N...C | 3.70 |
| Non-bonded | 4...2 | C...H | -0.93 |
| Non-bonded | 5...1 | N...H | -0.92 |
| Non-bonded | 5...2 | H...H | -2.06 |
| Non-bonded | 5...4 | C...H | -0.36 |
| Non-bonded | 6...1 | N...H | -1.07 |
| Non-bonded | 6...2 | H...H | -0.30 |
| Non-bonded | 6...4 | C...H | -0.42 |
| Non-bonded | 6...5 | H...H | -5.22 |
| Non-bonded | 7...1 | O...N | -0.58 |
| Non-bonded | 7...2 | O...H | -1.19 |
| Non-bonded | 7...3 | C...O | 5.40 |
| Non-bonded | 7...5 | O...H | -0.65 |
| Non-bonded | 7...6 | O...H | -0.88 |
| Non-bonded | 8...1 | O...N | 0.62 |
| Non-bonded | 8...2 | O...H | -0.37 |
| Non-bonded | 8...3 | C...O | 6.48 |
| Non-bonded | 8...5 | O...H | -3.53 |
| Non-bonded | 8...6 | O...H | -3.53 |
| Non-bonded | 8...7 | O...O | 11.60 |
| Non-bonded | 9...2 | H...H | -4.84 |
| Non-bonded | 9...3 | C...H | -1.05 |
| Non-bonded | 9...4 | C...H | -1.19 |
| Non-bonded | 9...5 | H...H | -0.28 |
| Non-bonded | 9...6 | H...H | -1.78 |
| Non-bonded | 9...7 | O...H | -2.57 |
| Non-bonded | 9...8 | O...H | -0.26 |
| Non-bonded | 10...1* | N...O | 1.20 |
| Non-bonded | 10...2* | O...H | -1.79 |
| Non-bonded | 10...3 | O...C | -0.20 |
| Non-bonded | 10...4 | O...C | 0.00 |
| Non-bonded | 10...5 | O...H | -0.25 |
| Non-bonded | 10...6 | O...H | -0.09 |
| Non-bonded | 10...7* | O...O | 2.75 |
| Non-bonded | 10...8 | O...O | -0.48 |
| Non-bonded | 10...9 | O...H | -0.32 |
| Non-bonded | 11...1 | H...N | -0.22 |
| Non-bonded | 11...2 | H...H | -0.28 |
| Non-bonded | 11...3 | O...C | -0.29 |
| Non-bonded | 11...4 | O...C | 0.07 |
| Non-bonded | 11...5 | H...H | 0.10 |
| Non-bonded | 11...6 | H...H | 0.09 |
| Non-bonded | 11...7* | H...O | -3.48 |
| Non-bonded | 11...8 | H...O | 0.28 |
| Non-bonded | 11...9 | H...H | -0.20 |
| Non-bonded | 12...1 | H...N | -0.16 |
| Non-bonded | 12...2 | H...H | 0.08 |
| Non-bonded | 12...3 | H...C | -0.07 |
| Non-bonded | 12...4 | H...C | -0.33 |
| Non-bonded | 12...5 | H...H | -0.26 |
| Non-bonded | 12...6 | H...H | -0.02 |
| Non-bonded | 12...7 | H...O | -0.19 |
| Non-bonded | 12...8 | H...O | -0.38 |
| Non-bonded | 12...9 | H...H | 0.11 |
| Non-bonded | 12...11 | H...H | -2.69 |
| Total Non-bonded |  |  | -14.01 |

*Interactions involved in hydrogen bonds.

**Table S2**. Electron correlation energies (ECEs) (kJ mol^-1^) for the ethene dimer.

| Type | Label | Atom or interaction | Correlation Energy (kJ mol^-1^) |
| --- | --- | --- | --- |
| Atomic | 1 | C | -413.93 |
| Atomic | 2 | C | -413.93 |
| Atomic | 3 | C | -413.76 |
| Atomic | 4 | C | -413.86 |
| Atomic | 5 | H | -35.48 |
| Atomic | 6 | H | -35.48 |
| Atomic | 7 | H | -35.48 |
| Atomic | 8 | H | -35.48 |
| Atomic | 9 | H | -35.45 |
| Atomic | 10 | H | -35.45 |
| Atomic | 11 | H | -35.45 |
| Atomic | 12 | H | -35.45 |
| Total Atomic |  |  | -1939.20 |
| Bond | 1-2 | C-C | 142.47 |
| Bond | 3-4 | C-C | 142.62 |
| Bond | 5-2 | C-H | 38.20 |
| Bond | 6-1 | C-H | 38.20 |
| Bond | 9-2 | C-H | 37.52 |
| Bond | 10-1 | C-H | 37.52 |
| Bond | 7-4 | C-H | 38.18 |
| Bond | 8-3 | C-H | 38.15 |
| Bond | 12-4 | C-H | 37.51 |
| Bond | 11-3 | C-H | 37.51 |
| Total bond | |  | 587.89 |
| Non-bonded | 5...1 | C...H | -1.32 |
| Non-bonded | 9...1 | C...H | -0.99 |
| Non-bonded | 6...2 | C...H | -1.32 |
| Non-bonded | 10...2 | C...H | -0.99 |
| Non-bonded | 7...3 | C...H | -1.36 |
| Non-bonded | 12...3 | C...H | -1.01 |
| Non-bonded | 6...4 | C...H | -1.33 |
| Non-bonded | 11...4 | C...H | -1.01 |
| Non-bonded | 9...5 | H...H | -4.23 |
| Non-bonded | 10...6 | H...H | -4.23 |
| Non-bonded | 12...7 | H...H | -4.23 |
| Non-bonded | 11...8 | H...H | -4.23 |
| Non-bonded | 6...5 | H...H | -2.83 |
| Non-bonded | 10...9 | H...H | -2.59 |
| Non-bonded | 12...11 | H...H | -2.59 |
| Non-bonded | 8....7 | H...H | -2.82 |
| Non-bonded | 9...6 | H...H | -1.03 |
| Non-bonded | 10...5 | H...H | -1.03 |
| Non-bonded | 11...7 | H...H | -1.03 |
| Non-bonded | 12...8 | H...H | -1.03 |
| Non-bonded | 11...9 | H...H | -0.68 |
| Non-bonded | 11...10 | H...H | -0.68 |
| Non-bonded | 9...11 | H...H | -0.68 |
| Non-bonded | 9...21 | H...H | -0.68 |
| Non-bonded | 10...4 | C...H | -0.35 |
| Non-bonded | 10...3 | C...H | -0.37 |
| Non-bonded | 9...4 | C...H | -0.35 |
| Non-bonded | 9...3 | C...H | -0.36 |
| Non-bonded | 11...1 | C...H | -0.35 |
| Non-bonded | 11...2 | C...H | -0.35 |
| Non-bonded | 12...1 | C...H | -0.32 |
| Non-bonded | 12...2 | C...H | -0.33 |
| Non-bonded | 3...1 | C...C | 0.36 |
| Non-bonded | 3...2 | C...C | 0.37 |
| Non-bonded | 4...1 | C...C | 0.34 |
| Non-bonded | 4...2 | C...C | 0.34 |
| Non-bonded | 11...5 | H...H | 0.21 |
| Non-bonded | 11...6 | H...H | 0.21 |
| Non-bonded | 12...5 | H...H | 0.21 |
| Non-bonded | 12...6 | H...H | 0.21 |
| Non-bonded | 3...5 | C...H | -0.01 |
| Non-bonded | 3...6 | C...H | -0.01 |
| Non-bonded | 4...5 | C...H | -0.01 |
| Non-bonded | 4...6 | C...H | -0.01 |
| Non-bonded | 8...9 | H...H | 0.21 |
| Non-bonded | 8...10 | H...H | 0.21 |
| Non-bonded | 7...9 | H...H | 0.21 |
| Non-bonded | 7...10 | H...H | 0.21 |
| Non-bonded | 7...2 | C...H | -0.01 |
| Non-bonded | 7...1 | C...H | -0.01 |
| Non-bonded | 8...2 | C...H | -0.01 |
| Non-bonded | 8...1 | C...H | -0.01 |
| Non-bonded | 8...5 | H...H | -0.17 |
| Non-bonded | 8...6 | H...H | -0.17 |
| Total non-bond | |  | -43.98 |
